# Make Headway for Echinococcosis: Take along the Ignored Cases

**Published:** 2019

**Authors:** Aisha KHAN, Haroon AHMED, Sami SIMSEK

**Affiliations:** 1. Department of Biosciences, COMSATS University Islamabad (CUI), Islamabad, Pakistan; 2. Department of Parasitology, Faculty of Veterinary, University of Firat, Elazig, Turkey

## Dear Editor-in-Chief

Cystic echinococcosis (CE) is endemic in many regions of Central Asia putting up almost 58% of population at risk including areas of Mongolia, Kazakhstan, Kyrgyzstan, Tajikistan, Turkmenistan, Uzbekistan, Afghanistan, Iran, Pakistan and western China (1). Pakistan is considered to be endemic region for CE and more elaborate work is still required (2). In order to know the true nature of endemicity of this neglected disease, the generation of epidemiological data is essential. Lack of resources hinders the diagnosis and characterization of disease which in turn makes control and prevention difficult.

Cystic Echinococcosis (CE) is responsible for over US$ 3 billion in expenses globally every year (3). It is more prevalent in areas where people survive on animal husbandry and agricultural activities (4), and the rate is higher in nomadic and semi-nomadic populations due to their lifestyle (5). One of the major risk factors for CE is open slaughtering of livestock without any veterinary supervision. Previous published data on slaughtered animals showed that 22.6 million livestock were slaughtered in Pakistan. Approximately 3.56 million cattle, 3.34 million buffaloes, 4.74 million sheep and 11.0 million goats were slaughtered in 2006 (6). Owing to open slaughtering practice, infected offals are ingested by stray dogs (definitive host), and eggs are spreads to the environment (2).

In Pakistan, lack of research data at molecular and epidemiological level of CE necessitates more focus on this area of research. To best of our knowledge, published data to date show 1611 CE cases in humans from different cities of Pakistan. These published reports are mostly based on data obtained from different areas of the country (7). There is no surveillance system to record the reported cases of CE. Therefore, it can be assumed that actual occurrence of CE might have been many folds of cases reported across the country. In some countries estimates based on hospital records might underestimate the actual value by 700 fold (8).

Limited epidemiological data on CE impedes in devising effective management strategies for its control. Recently a useful ultra-sound-based study of latent CE and AE cases in rural parts of Romania, Bulgaria, and Turkey (n=119/24,681) was reported which adds to the knowledge of global epidemiology of these diseases (8). The burden of CE in rural areas is usually estimated by using available hospital record. However due to a large number of sub clinical cases hospital reports do not reflect real prevalence data. Cross-sectional studies based on population surveys using ultrasound technique would provide more valid data. The control and prevention strategy depends upon the endemicity of disease defined by epidemiological data (8).

In China, in an effort to generate global database for effective control of echinococcosis, the Belt and Road Network for the Elimination and Control of Echinococcosis and Cysticercosis (B & R-NEC) consisting of 13 countries was established in Chengdu, China in 2017 (9), but unfortunately Pakistan was not included in the list. Recently the process of Pakistan to become a part of this alliance have been initiated (Personal communication).

Most of the population in Pakistan lives in rural area with unhygienic life styles. If such increasing burden of the disease is left un-checked, it would pose greater threat to animal and public health. The international research cooperation is a needed act of time to combat and root out this silently evolving neglected tropical disease. The seriousness of issue needs to be addressed by international community to make a point for effective control strategies on regional and global level.
